# Mechanisms of survival, responses and sources of *Salmonella* in low-moisture environments

**DOI:** 10.3389/fmicb.2013.00331

**Published:** 2013-11-14

**Authors:** Sarah Finn, Orla Condell, Peter McClure, Alejandro Amézquita, Séamus Fanning

**Affiliations:** ^1^UCD Centre for Food Safety, School of Public Health, Physiotherapy and Population Science, University College DublinBelfield, Dublin 4, Ireland; ^2^Safety and Environmental Assurance Centre, Unilever, Colworth Science ParkSharnbrook, Bedfordshire, UK

**Keywords:** low-moisture, *Salmonella*, survival, phenotypes, adaptation

## Abstract

Some Enterobacteriaceae possess the ability to survive in low-moisture environments for extended periods of time. Many of the reported food-borne outbreaks associated with low-moisture foods involve *Salmonella* contamination. The control of *Salmonella* in low-moisture foods and their production environments represents a significant challenge for all food manufacturers. This review summarizes the current state of knowledge with respect to *Salmonella* survival in intermediate- and low-moisture food matrices and their production environments. The mechanisms utilized by this bacterium to ensure their survival in these dry conditions remain to be fully elucidated, however, in depth transcriptomic data is now beginning to emerge regarding this observation. Earlier research work described the effect(s) that low-moisture can exert on the long-term persistence and heat tolerance of *Salmonella*, however, data are also now available highlighting the potential cross-tolerance to other stressors including commonly used microbicidal agents. Sources and potential control measures to reduce the risk of contamination will be explored. By extending our understanding of these geno- and phenotypes, we may be able to exploit them to improve food safety and protect public health.

## INTRODUCTION

Drying is a traditional method that has been used to preserve food and to this day low-moisture foods have constituted a substantial part of our diet. Foods in this category have a long shelf life and are usually stable for years. Low- and intermediate-moisture foods have a reduced water activity (*a*_w_). The term *a*_w_ was originally applied by the pharmaceutical and food industries as a quantitative measure used in the determination of the shelf life of a product. *a*_w_ can be defined as the ratio of the vapor pressure of water in a food matrix compared to that of pure water at the same temperature (Labuza, 1980). Pure distilled water has an *a*_w_ of 1 and low-moisture products have a reduced value relative to this. The *a*_w_ of a product is also dependant on factors such as storage temperature and composition. Examples of products naturally low in moisture include nuts, cereals, and honey. Other low-moisture foods could be high-moisture products that have been subjected to a drying process, such as powdered infant formula (PIF). Other examples include dried fruit and fruit conserves, soup mixes, milk-based powders, preserved meat and fish, chocolate, peanut butter, pasta, herbs and spices, grains and seeds, and animal feeds. Although sometimes erroneously believed to be low risk because they are unable to support microbial growth, all of these food matrices remain susceptible to microbial contamination and may pose a risk to consumers. This misconception can lead to manufacturers releasing unsafe products and also inappropriate preparation practices that can render the product unsafe for consumption due to microbial proliferation. An example of the latter includes the storing of reconstituted PIF at room temperature for extended periods of time (Beuchat et al., 2011). The *a*_w_ value can give manufacturers an indication of the susceptibility of their product to microbial growth, as well as an indication of the types of microorganisms that may proliferate under these conditions.

The availability of water for biological reactions can be reduced by a number of methods such as freezing, the physical removal of water (such as spray drying), or by the addition of solutes such as salt and sugar (Brown, 1976). Although reducing the *a*_w_ of food is undoubtedly a useful preservation technique, many microorganisms can survive this process. Bacterial metabolism may be significantly reduced but spores and vegetative cells can remain viable for months to years in dried foods and also in the abiotic fabric of the corresponding production facilities. In addition, contamination of low-moisture foods can come about from exposure to the processing environment following a lethal intervention step (such as thermal treatment) or through addition of poor quality ingredients after an intervention step. For some pathogens, such as *Salmonella*, presence of low numbers in a low-moisture food poses a risk even though growth is prevented. For low infective dose microorganisms, these must not be present at any level in a food just prior to consumption.

Once a bacterium has contaminated a dry-food production environment, its subsequent removal can prove challenging. One of the most significant risk factors for *Salmonella* contamination in dry processing environments is presence of water, which allows growth and spread of the organism in the environment thereby increasing the risk of product contamination. In these processing environments the use of wet cleaning should be restricted due to the risk it poses, and only used when it is considered essential, as in the case of product contamination incidents. In addition, other methods used to reduce microorganisms in high-moisture foods such as mild heat treatment and high pressure cannot be used in the same way for low-moisture foods (Beuchat et al., 2011).

Ready-to-eat (RTE) foods are those that do not require any further processing (such as cooking) prior to consumption by the consumer. Following the purchase of these commodities, there is no subsequent critical step applied to eliminate any pathogenic bacteria that may be present. Examples of low-moisture RTE foods include chocolate and peanut butter. If a product is intended to be cooked before eating, it is necessary for producers to outline the appropriate cooking procedure to be used by the consumer. This process should take into account the added heat tolerance of pathogens within low-moisture products relative to those present in high-moisture foods if the foods are not rehydrated prior to cooking. The cooking process should also be validated by the manufacturer. Despite these strategies, however, there is no guarantee that the consumer will adhere to these instructions, therefore further measures should be implemented by the manufacturer to ensure the elimination of pathogens in the food product prior to its distribution (Beuchat et al., 2011, 2013). In considering this scenario, producers may be required, for food safety considerations, to classify certain products as RTE although they are intended to be cooked before consumption, if that product is in fact eaten raw on a common basis by the population. In these cases it is also essential to ensure the best quality ingredients are used in order to further reduce the chance of contamination and to consider using altered labeling to deter the consumer from consuming the product in its raw state (Trybus and Johnson, 2010). An example of this can be illustrated by the outbreak of *Escherichia coli* O157:H7 that was associated with the consumption of a raw cookie dough product that occurred in the United States in 2009. This product was consumed directly from refrigeration without the cooking step that would normally be required. Producers promptly dealt with this issue and were able to resume production later that same year (Trybus and Johnson, 2010).

The optimum *a*_w_ for growth of most microorganisms is in the range of 0.995–0.98 (Lund et al., 2000). In the context of pathogenic food-borne bacteria the best adapted of these is *Staphylococcus aureus* requiring a minimum *a*_w_ for growth of approximately 0.85, with increased moisture levels required for toxin production (Brown, 1976; Notermans and Heuvelman, 1983). The minimum *a*_w_ for the majority of bacteria is 0.88–0.91 (Farkas et al., 2007), however, food-borne pathogens can survive for extended periods in products at an *a*_w_ < 0.85 (Carrasco et al., 2012). Gram-negative bacteria require an *a*_w_ > 0.93 for growth, and this value is also relevant for *Salmonella* (D’Aoust et al., 1997).

## *Salmonella* SURVIVAL IN LOW-MOISTURE FOODS AND ENVIRONMENTS

*Salmonella* are one of the most challenging bacteria for food manufacturers, and are a major cause of gastroenteritis. It is estimated that 93.8 million cases of salmonellosis occur globally each year, with 80.3 million of these being attributed to the consumption of contaminated food products (Majowicz et al., 2010). Furthermore, an estimated 155,000 deaths are reported annually due to *Salmonella* infection (Majowicz et al., 2010). The majority of reported food-borne illness outbreaks related to low-moisture products occur as a result of *Salmonella* contamination (**Table 1**). This pathogen is prevalent in raw ingredients and can survive under harsh, dry conditions for lengthy periods of time (Podolak et al., 2010; Van Doren et al., 2013). The survival of this bacterium is not only dependant on the *a*_w_ of the environment or food but also on other factors such as matrix composition and storage temperature. The main causes of *Salmonella* contamination in low-moisture foods are poor sanitation practices, substandard facilities, equipment design, and improper maintenance (Carrasco et al., 2012). A selection of *Salmonella* survival studies related to low moisture are described below.

**Table 1:** List of selected outbreaks of *Salmonella* infection after consumption of low-moisture foods.

Year	*Salmonella* serotype(s)	Product	Location	Number of people affected	Reference
1973	Derby	Powdered milk	Trinidad	3000	Weissman et al. (1977)
1974	Eastbourne	Chocolate	Canada	95	D’Aoust et al. (1975)
1982	Napoli	Chocolate	UK	245	Gill et al. (1983)
1985	Ealing	Powdered infant formula	UK	76	Rowe et al. (1987)
1987	Typhimurium	Chocolate	Norway, Finland	361	Kapperud et al. (1990)
1993	Rubislaw, Saintpaul, and Javiana	Potato chips	Germany	1000	Lehmacher et al. (1995)
1995	Senftenberg	Infant food	UK	5	Rushdy et al. (1998)
1996	Enteritidis PT4	Marshmallow	UK	45	Lewis et al. (1996)
1996	Mbandaka	Peanut butter	Australia	15	Scheil et al. (1998)
1998	Agona	Cereal	USA	209	CDC (1998)
2000	Enteritidis PT30	Almonds	USA, Canada	168	Isaacs et al. (2005)
2001	Oranienburg	Chocolate	Germany	439	Werber et al. (2005)
2001	Stanley and Newport	Peanuts	Australia, Canada, UK	109	Kirk et al. (2004)
2003	Agona	Tea	Germany	42	Rabsch et al. (2005)
2005	Agona	Powdered infant formula	France	141	Brouard et al. (2007)
2006	Tennessee	Peanut butter	USA	628	CDC (2007b)
2008	Agona	Cereal	USA	28	CDC (2008)
2008	Typhimurium	Peanut butter	USA, Canada	714	Medus et al. (2009)
2009	Montevideo	Red and black pepper	USA	272	Julian et al. (2010)
2011	Enteritidis	Turkish pine nuts	USA	43	CDC (2011)
2012	Infantis	Dry dog food	USA	49	CDC (2012a)
2012	Bredeney	Peanut butter	USA	42	CDC (2012b)
2013	Montevideo/Mbandaka	Tahini past	USA	16	CDC (2013)

Spray-dried milk or egg powders are often used as ingredients in dry food production and these can pose a contamination risk for manufacturers (Jung and Beuchat, 1999; Cahill et al., 2008). A study conducted by Miller et al. (1972) examined the survival of *S.* Typhimurium during the spray drying process, with inlet air temperatures of 165 and 225°C and outlet air temperature of 67 and 93°C. It was noted that the total solids present prior to drying greatly affected levels of bacterial cell reduction achievable (Miller et al., 1972). Log reductions in *Salmonella* of between 6 and 3.3 were observed after spray drying of milk that contained 20 and 40% solids, respectively, thereby demonstrating that the more dense the product (and the greater the fat content) the greater the bacterial survival, likely due to the protective effect of the solids toward thermal inactivation (Miller et al., 1972). This study concluded that pasteurization of the milk prior to drying was an indispensable step, since *Salmonella* are easily destroyed in high-moisture ingredients but once drying has occurred the efficacy of this measure proves challenging (Miller et al., 1972). An additional study reported that survival of these pathogens in egg white powder maintained at 54°C for one week was enhanced by lowered moisture content (Jung and Beuchat, 1999). *Salmonella* are of foremost concern for the PIF industry and have been the cause of several illness outbreaks (Rowe et al., 1987; Brouard et al., 2007; Cahill et al., 2008). Barron and Forsythe (2007) compared the survival capabilities of several species of Enterobacteriaceae in PIF over a 2.5-year period. Interestingly, while *S.* Enteritidis showed similar survival capabilities to *E. coli* and *Klebsiella pneumoniae* (up to 15 months), they were out-survived by several other species (not judged to be major food-borne pathogens) including *Pantoea* spp. and *Escherichia vulneris*. However, *S.* Enteritidis were also out-survived by the neonatal pathogen *Cronobacter*, whereby some capsular strains were still recoverable after 2.5 years (Barron and Forsythe, 2007).

Storage of a product under vacuum or air, and at various temperatures, can affect pathogen survival, and predicting the reduction of salmonellae cannot be done on the basis of *a*_w_ alone (Kotzekidou, 1998). This fact was demonstrated in a study where the survival of *S. *Enteritidis, within refrigerated, vacuum packed halva, a sesame seed-based product with an *a*_w_ of 0.18, was documented over an 8-month period (Kotzekidou, 1998). This study demonstrated increased survival of *Salmonella* within a vacuum packed product in comparison to air-sealed packaging, with slightly better recovery after 8 months from halva stored at 4°C compared to room temperature storage (Kotzekidou, 1998).

Another popular low-moisture food product, peanut butter (*a*_w_ 0.45–0.2), has also been implicated in a number of *Salmonella* outbreaks (Burnett et al., 2000; Ma et al., 2009; Medus et al., 2009; CDC, 2012b). This food product is comprised of a colloidal suspension of lipid and water within a peanut meal phase. Bacterial cells inoculated into this matrix generally aggregate within, or in proximity, to the water phase and as a result the survival of cells may be affected by the size of water and lipid droplets in the meal phase. The cell density within these droplets also affects nutrient availability (Burnett et al., 2000). *Salmonella* have been shown to survive better in peanut butter at higher numbers when stored at a temperature of 4°C compared to 21°C (Burnett et al., 2000). These observations suggest that if high temperatures used during production are inadequate to reliably reduce *Salmonella* cell numbers or if recontamination/cross-contamination are possible, then prolonged survival in this product is a likely outcome (Carrasco et al., 2012; Nummer and Smith, 2012).

Chocolate is one of the most popular RTE products consumed worldwide and has been the vehicle in a number of salmonellosis outbreaks. In general it has a moisture content <8%, corresponding to an *a*_w_ of 0.5–0.4 (Werber et al., 2005). Control of *Salmonella* is a challenging task for chocolate manufacturers due to several factors outlined below. The combined effect of low-*a*_w_ along with high fat content increases the thermal tolerance of *Salmonella*, therefore temperatures used during production may not kill this pathogen. Further, *Salmonella* contamination of the raw ingredients, such as milk powder or cocoa beans, can also occur (Werber et al., 2005). With *Salmonella*, a low number of cells may be sufficient to cause illness and a large number of people, over a wide geographical region could be affected (Werber et al., 2005). It has previously been documented that in certain salmonellosis outbreaks, very low numbers of cells were present in the contaminated product, in the range of 13 CFU/g. This fact suggested that a low infectious dose, between 10 and 1,000 cells, was associated with *Salmonella* and cases of illness (Greenwood and Hooper, 1983; Kapperud et al., 1990; Werber et al., 2005). Further, clumping of bacteria within the food matrix, due to a non-homogeneous distribution, could lead to an erroneous underestimation of infective dose and consequently when ingested in this clumped state, have the potential to cause illness (Kapperud et al., 1990; Werber et al., 2005). Alternatively the high fat content of the chocolate may protect the bacterial cells against gastric acid in the stomach, thereby allowing the cells to enter and colonize the lower gastrointestinal (GI) tract (D’Aoust, 1977). Tamminga et al. (1976) conducted a study examining survival of *S.* Eastbourne and *S.* Typhimurium in inoculated chocolate at room temperature. The *S.* Eastbourne isolate was still recoverable even after 19 months of storage, however, *S.* Typhimurium showed a greater rate of decline and became unrecoverable after 15 months of storage (Tamminga et al., 1976). However, as this study examined only one strain of each serotype it is unclear whether results observed would be strain or serotype dependant. Similar survival within inoculated chocolate has been observed for a selection of *E. coli* strains, monitored for a period of 12 months (Baylis et al., 2004). This may suggest that *Salmonella* do not possess any greater capacity for survival in chocolate in comparison to other bacteria, such as *E. coli*. This could then mean that the observation that *Salmonella* are the causative agent of the majority of chocolate related outbreaks of disease is attributed to the prevalence of this pathogen in raw ingredients and the environment rather than a greater ability to survive over other bacterial species.

*Salmonella* have the potential to survive for long periods of time in a desiccated state, on work surfaces and equipment, as well as in food matrices. For example, Gruzdev and Pinto (2012) demonstrated that *Salmonella* dehydrated on a plastic surface can survive for more than 100 weeks under refrigeration. Similarly, Hiramatsu et al. (2005) simulated desiccation on an abiotic surface by drying a selection of *Salmonella* isolates onto paper disks. Following an initial reduction in bacterial cell numbers within the first 24-h period, *Salmonella* were detected for up to 35 and 70 days when stored at 35 and 25°C, respectively. Interestingly, numbers remained constant for up to 24 months when stored at 4°C. These findings once again highlight the importance of storage temperature on survival and suggest that storing contaminated foods in a refrigerator may have serious food safety consequences (Hiramatsu et al., 2005). Increasing the levels of sucrose in the disks further enhanced survival by up to 79-fold, conversely, an increase in NaCl content decreased the number of surviving bacteria, a feature that emphasizes the impact of solute identity on survival (Hiramatsu et al., 2005). Eriksson de Rezende et al. (2001) postulated that *in vitro* adaptation of *Salmonella* may occur as a result of exposure to alternating levels of high- and low-*a*_w_ and that an increased tolerance may result from a combination of biofilm formation, the entry into a dormant state and physicochemical changes within the organism. Such fluctuations between high- and low-*a*_w_ could occur following a wet cleaning routine and the subsequent drying of production area; the cycling of *a*_w_ perhaps promoting survival of the bacteria.

It is clear that the survival capabilities of *Salmonella* can be serotype dependent and the period of survival is influenced by several factors such as temperature, solid content, and the food matrix itself.

## RESPONSES AND MECHANISMS FOR SURVIVAL IN LOW-MOISTURE CONDITIONS

**FIGURE 1:**
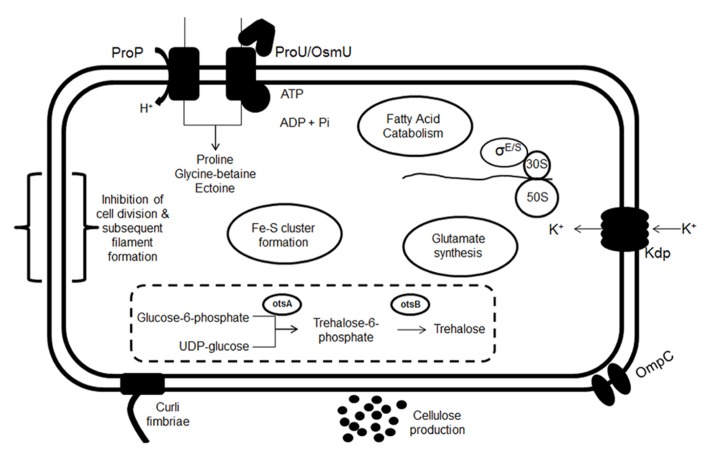
**Summary of proposed responses occurring upon transition of a bacterial cell into a low-moisture environment.** These include K^+^ uptake by the Kdp transporter, osmoprotectant transport (ProU, ProP, and OsmU), glutamate synthesis and trehalose biosynthesis. The up-regulation of fatty acid catabolism, Fe–S clusters formation and filament formation have also been observed, as well as an up-regulation of the RpoE and RpoS regulators. An increase in the number of OmpC porins in reduced moisture has also been observed. Finally there may be a possible role for cellulose and curli fimbriae in survival.

Although numerous studies have been carried out investigating the phenotypes associated with *Salmonella* in low-moisture conditions, particularly with regards to increased heat tolerance, the mechanism(s) by which these bacteria are able to survive such harsh conditions are somewhat less well understood. In low-moisture conditions, bacterial cells attempt to maintain their turgor pressure by an increase in the intracellular concentration of compatible solutes. The response of bacteria involve an immediate response to quickly balance osmotic pressure, such as an influx of K^+^, followed by a longer term adaptation, for instance the uptake of compatible solutes (Csonka, 1989). A summary of responses/survival mechanisms is schematically shown in **Figure 1** and discussed below.

### OSMOPROTECTANTS

When bacteria are exposed to a low-*a*_w_ environment, they must balance the osmolarity of their internal cell composition with that of the external environment in order to avoid the loss of water. Bacteria possess numerous cellular mechanisms that are involved in this process of osmoregulation. As an example, the accumulation of electrically neutral, low molecular weight compatible solutes (osmoprotectants), such as proline, glycine-betaine, and ectoine can facilitate the bacterial cell to limit the loss of water (Csonka and Hanson, 1991). Osmoprotectants can concentrate to high levels within the bacterial cell without affecting enzyme function (Csonka, 1989). The main transporters in question are ProP [a member of major facilitator superfamily (MFS) permeases] and ProU and OsmU (both ABC-transporters), all of which have been shown to play important roles under low-*a*_w_ stress in a liquid system (**Figure 1**; Cairney et al., 1985a,b; Stirling et al., 1989; Frossard et al., 2012). Recently, an up-regulation of the *proU* and *osmU* genes have been documented in two studies investigating the transcriptomic changes occurring during the early stages of desiccation in *Salmonella* (Li et al., 2012; Finn et al., 2013a). Finn et al. (2013a) also highlighted the critical importance of *proP*, as mutants lacking this gene demonstrated a reduced long-term desiccation survival on a stainless steel surface.

The biosynthesis of the disaccharide trehalose, is another compatible solute that is also important for osmoadaptation in *Salmonella* (Csonka and Hanson, 1991; Kempf and Bremer, 1998; Balaji et al., 2005; Strom and Kaasen, 2006). Trehalose is produced by the enzymatic condensation of glucose-6-phosphate and UDP-glucose by trehalose-6-phosphate synthase (OtsA), followed by the formation of trehalose from the resulting intermediate by trehalose-6-phosphate phosphatase (OtsB), and is dependent on the alternative sigma factor RpoS for the induction of *otsAB* (**Figure 1**; Kempf and Bremer, 1998). The *otsB* gene has been shown to be induced in *Salmonella* 6 min after NaCl shock (Balaji et al., 2005). An up-regulation in the trehalose biosynthetic genes has also been observed after desiccation of *Salmonella* on paper disks and stainless steel (Li et al., 2012; Finn et al., 2013a). As glucose is diverted toward trehalose production, cells must acquire energy for cellular processes (such as the import of osmoprotectants) from an alternative source. It has been proposed that cells derive this energy by fatty acid catabolism which is a very-cost effective energy source due to the production of more ATP per carbon atom (from fatty acids in comparison to glucose). In line with this, fatty acid catabolic genes were found to be up-regulated under desiccation stress in aerobic conditions (Li et al., 2012; Finn et al., 2013a). It is important to note that transcriptomic responses observed toward desiccation are highly dependent on the environmental conditions in question. For example, both the Li et al. (2012) and Finn et al. (2013a) studies were carried out at room temperature, under aerobic conditions, and it is likely that an alternative set of differentially expressed genes would be identified under desiccation stress in altered temperature or oxygen levels. An up-regulation in genes involved in the formation of Fe–S clusters has also been detected upon desiccation and this may also be linked to an increased energy mandate, then being provided *via* the terminal electron transport chain (Finn et al., 2013a). Investigating the mechanisms involved in desiccation tolerance is a relatively new area of research and whether the same systems described above also play a role in bacterial survival in a factory setting, remain to be described.

It was previously thought that the production or transport of osmoprotectant molecules was dependant on an initial increase of K^+^ and its counterion glutamate in the cell (**Figure 1**; Booth and Higgins, 1990). Since glutamate can then have a knock-on inhibitory effect on cellular functions, it was hypothesized that in an effort to avoid this, potassium glutamate stimulates the accumulation of osmoprotectants (Epstein, 1986; Csonka, 1989; Booth and Higgins, 1990; Lee and Gralla, 2004). Other models predict that the ion concentration within the cell directly controls the osmotic response (Balaji et al., 2005). Using qRT-PCR to monitor osmoregulated genes in *S.* Typhimurium, Balaji et al. (2005) reported that a different series of events may occur. In the latter study the first genes determined to be induced in response to NaCl were those involved in the transport of proline, betaine, and other osmoprotectants, as well as the alternative sigma factor RpoS, denoted as σ^ S^ (Balaji et al., 2005). The mechanisms used by *Salmonella* to counteract an osmotic imbalance were also dependent on the chemical nature of the solute. For example, the *kdp* genes encoding a K^+^ transporter were found to be induced at a level of 170-fold higher when cells were exposed to 0.3 M NaCl in comparison to 0.6 M sucrose (Balaji et al., 2005).

### ALTERNATIVE SIGMA FACTORS, rRNA DEGRADATION, AND VBNC STATE

Both σ^ S ^ and σ^ E ^ have been linked with survival during starvation and osmotic stress conditions, but their relative importance may depend on the environmental composition (McMeechan et al., 2007). The alternative sigma factor σ^E^ has also been determined as important for dehydration tolerance in *Salmonella*, since Δ*rpoE* mutants are severely compromised in their long-term desiccation survival capacity in comparison to the wild-type (Gruzdev et al., 2012; Finn et al., 2013a). In *E. coli*, an increase in negative supercoiling of DNA appears to be necessary for the induction of a subset of genes critical for osmotic response, and these included *rpoS* (Cheung et al., 2003). DNA supercoiling has also been suggested to play a regulatory role in the induction of *proU*, an ABC-transporter involved in the accumulation of osmoprotectants in *S.* Typhimurium (Higgins et al., 1988). 

Deng et al. (2012) conducted a study investigating the transcriptome of *S.* Enteritidis in peanut oil (*a*_w_ 0.3) using RNAseq. This was one of the first studies to examine the bacterial response in a low-*a*_w_ food matrix and provided an initial insight into the mechanisms employed for survival. Results showed that <5% of the genome was transcribed in the peanut oil compared to 78%, when the same bacteria was cultured in LB broth (Deng et al., 2012). It would therefore seem that *Salmonella* enters a partially dormant state as has been documented in other cases of starvation stress (Lewis, 2006). An increased level of rRNA degradation, as was observed in this study, may serve as a possible nutrient source for the stressed bacteria (Deutscher, 2006; Deng et al., 2012). Similar groups of genes were found to be up-regulated after 22 h of desiccation on a plastic surface in a recent study by Gruzdev et al. (2012). In the latter study, 90 genes were up-regulated, including those involved in ribosomal structure, amino acid metabolism, stress response, and ion transport. Genes encoding a potassium transport channel, *kdpFABC*, were amongst those highly up-regulated, however, it was later observed that a mutation within this operon did not affect desiccation tolerance but did compromise long-term cold storage in a dehydrated state (Gruzdev et al., 2012). As both the study by Deng et al. (2012) and Gruzdev et al. (2012) were carried out after extended periods of desiccation, the signals observed may also reflect variances in the degradation of RNA already present at the point of desiccation rather than the production of new mRNA. This may account for the absence of those genes originally present earlier during the desiccation process (Finn et al., 2013a).

A viable but non-culturable (VBNC) state has been reported for numerous pathogenic bacteria, including *Salmonella*, in response to stress (Oliver, 2010). In this case, the bacteria are thought to enter a metabolically dormant state and consequently are not culturable using conventional laboratory protocols. Nonetheless, VBNC bacteria retain their viability and upon resuscitation under favorable conditions, bacterial growth is restored (Gupte et al., 2003). VBNC *Salmonella* have been found in both low-*a*_w_ media and under desiccation stress and may present another method for long-term survival (Eriksson de Rezende et al., 2001; Gruzdev and Pinto, 2012).

### FILAMENTATION

Mattick et al. (2000b) demonstrated that growth under sub-optimal *a*_w_ conditions where NaCl, glycerol, or sucrose was used as the humectant, induced the formation of filaments in all the *Salmonella* isolates studied, after 144 h of incubation. Culture media supplemented with NaCl (*a*_w_ between 0.98 and 0.95) were identified as the optimal growth condition for filament formation (Mattick et al., 2000b). Filament formation may occur due to the inhibition of cell division proteins as a result of osmotic stress. However, these authors hypothesized that *Salmonella* may produce inhibitors of cell division in response to osmotic stress in order to gain a survival advantage (Mattick et al., 2000b). This phenotype has also been observed in other *Salmonella* serotypes in response to low-*a*_w_ (Shaw, 1968; Eriksson de Rezende et al., 2001; Kieboom et al., 2006). The formation of filaments leads to an increase in overall biomass without any increase in cell numbers. Naturally, this presents a problem for the food manufacturers. If bacterial filamentation occurs within a food product it could lead to the underestimation of potential cell numbers present; as the formation of long filaments will not increase the CFU when tested using conventional microbiological methods. Use of an enrichment step, however, may allow septation to occur resulting in higher cell numbers that can be enumerated. The formation of filaments prior to entrance into a dried state has been shown to lead to increased desiccation tolerance in comparison to non-filamentous cells on a stainless steel surface (Stackhouse et al., 2012). These data may suggest a role for filaments in persistence within a factory setting, whereby exposure to sub-optimal *a*_w_ levels, perhaps upon transition from “wet” to “dry” areas of the factory, that induce filament formation prior to drying within the environment may actually lead to increased survival. However, the possibility of this occurring within the production environment would require further investigation.

### OUTER MEMBRANE PORINS

In response to low-*a*_w_ stress, the expression of two outer membrane porins, OmpF and OmpC, is altered *via* regulation exerted by the two-component regulator EnvZ/OmpR (Hall and Silhavy, 1981a,b; Feng et al., 2003; Wang et al., 2012). These porins are involved in the passive diffusion of osmoprotectants in both *Salmonella* and *E. coli* (Kempf and Bremer, 1998). OmpF is the more predominant porin expressed at low osmolarity. The EnvZ sensor kinase can detect an increase in osmolarity resulting in higher concentrations of phosphorylated form of OmpR. This in turn results in an up-regulation of *ompC* with OmpC becoming the more predominant porin (Feng et al., 2003). OmpF is also post-translationally negatively regulated by the antisense RNA MicF (Pratt et al., 1996). An increase in *ompC* transcripts has been found to occur 12 min after a NaCl shock in *Salmonella* (Balaji et al., 2005). Hence, this switch to an OmpC predominant phenotype may prove important for the adaptation of the bacteria to low-*a*_w_ foods where NaCl is the principal humectant. Interestingly, it does not appear that this shift occurs upon desiccation (Deng et al., 2012; Gruzdev et al., 2012; Li et al., 2012; Finn et al., 2013a). However, if *ompC* mRNA has a short half-life, it is possible that these signals may be missed in a desiccation system due to RNA degradation.

### BIOFILM FORMATION

It is well known that *Salmonella* can form biofilms under numerous conditions and in response to starvation stress (Solano et al., 2002). It is possible therefore, that biofilm formation may play a role in the survival of *Salmonella* in response to desiccation and low-*a*_w_ stress. Production of curli fimbriae, one of the main components of biofilms, and cellulose have both been shown to enhance long-term desiccation survival (**Figure 1**; White et al., 2006; Vestby et al., 2009). However, in a non-dry environment one study demonstrated the presence of curli fimbriae is not important in persistence on conveyor belts, instead surface type may be a more influential factor (Stocki et al., 2007). No up-regulation of the biosynthetic genes involved have been observed in two studies investigating the transcriptome of desiccated *Salmonella* (Li et al., 2012; Finn et al., 2013a). The production of glycocalyx layers (composed of exopolysaccharides and proteins) that form a protective gel type extracellular matrix have also been shown to have a protective effect on bacteria against desiccation stress (Ophir and Gutnick, 1994; Spector and Kenyon, 2012). Nonetheless, whether biofilm production has an integral role in low-*a*_w_ survival remains to be determined.

The majority of studies investigating osmotic responses use NaCl as a model system, however, in the food industry many other humectants are used both in isolation and in combination. Due to concerns arising from the human health implications of NaCl in the diet, other humectants have been attracting attention. As such, it is possible that bacteria exhibit other as yet unknown responses to ensure their long-term survival within an actual food product.

## PHENOTYPES ASSOCIATED WITH SURVIVAL IN LOW-MOISTURE ENVIRONMENTS

A number of phenotypes have been associated with *Salmonella* isolated from low-*a*_w_ environments. Some of the phenotypes linked to exposure to a low-*a*_w_ environment are described, including the reduction in infectious dose, a noted increase in heat tolerance and cross-tolerance to other stressors.

### LOW INFECTIOUS DOSE

Salmonellosis that is epidemiologically linked to the ingestion of a contaminated low-*a*_w_ product may arise from a low infectious dose (of the order 10–100 CFU). This contrasts with the infectious dose following ingestion of other contaminated foods (>10^5^ CFU; Greenwood and Hooper, 1983; Rowe et al., 1987; Kothary and Babu, 2001; Todd et al., 2008). At present, the reason(s) underlying this observation remain to be completely elucidated. It could be due a non-homogeneous distribution or clumping of the infectious agent within the food matrix which may in turn lead to the underestimation of actual numbers of bacteria contaminating the low-a_w_ food matrix (Kapperud et al., 1990; Werber et al., 2005). The nature of the food matrix itself will also offer protective properties that allow for the safe transit of bacteria through the GI tract (D’Aoust, 1977; Todd et al., 2008). A recent study reported by Aviles et al. (2013) demonstrated this fact, showing that the combination of a high fat, low-*a*_w_ peanut butter matrix provided protection to *S*. Tennessee transiting through a simulated GI tract. Also, as mentioned above, a low infectious dose has been documented in relation to chocolate related food-borne illness outbreaks (Greenwood and Hooper, 1983; Werber et al., 2005). The use of chocolate as a vehicle for probiotic delivery to the colon has shown to be effective, with high survival levels of *Lactobacillus helveticus* and *Bifidobacterium longum* (Possemiers et al., 2010). The nature of the chocolate matrix offers a protective environment for transition through the GI tract, therefore the low infectious dose observed for *Salmonella* within this product may be directly related to the chocolate environment, rather than an increase in pathogenicity.

As mentioned above, *Salmonella* have the ability to form filaments under moderately low-*a*_w_ conditions and upon rehydration and regrowth, can achieve high bacterial loads within a relatively short period of time (Mattick et al., 2000b; Stackhouse et al., 2012). As a consequence, whilst only low levels of contamination are being detected, high-numbers of the pathogen could be ingested. If filamentous cells formed at intermediate moisture levels can then contaminate the finished product it would be of particular concern in the case of PIF which is often rehydrated and maintained at room temperature for extended periods of time, a practice that does not comply with World Health Organisation (WHO) safety guidelines and which allows sufficient time for a low number of contaminating cells to proliferate (Cahill et al., 2008). Bacterial cells in filamentous form, have given rise to an underestimation of the true number of contaminating cells whilst retaining their virulence phenotype *in vitro* and *in vivo* (Stackhouse et al., 2012). These filamentous bacteria also appear to have an increased tolerance to low pH (as determined following a 10 min exposure to pH 2 when compared with planktonic cells) and the ability to grow in 10% bile salts after a 24-h period of exposure, possibly selecting for a survival advantage when transiting through the GI tract of the host (Stackhouse et al., 2012).

In addition, entry into a VBNC state may also explain why only low numbers of bacteria are detectable within low-*a*_w_ foods (Oliver, 2010; Gruzdev and Pinto, 2012). Importantly, some studies have reported that cells resuscitated from a VBNC state can retain their pathogenic capacity (Caro et al., 1999; Lesne et al., 2000; Oliver, 2010).

### CROSS-PROTECTION TOWARD OTHER STRESSORS

Exposing *Salmonella* to low-*a*_w_ environments can provide this pathogen with a degree of cross-protection to other common stresses encountered in the production environment and during infection. For example, filamentous cells produced in response to low-*a*_w_ conditions have been shown to display a higher resistance to disinfectants commonly used in the food industry including sodium hypochlorite (Kieboom et al., 2006; Stackhouse et al., 2012). When exposed to pH 2 for between 5 and 10 min, filaments exhibited an increased survival (Stackhouse et al., 2012). Stackhouse et al. (2012) reported that when filaments were exposed to 10% bile salts, there was a significant (*P* < 0.01) decrease in survival when compared to planktonic controls after 4 h of exposure. Conversely, after 24 h of exposure to the same concentration of bile salts the growth of filamentous cells compared to control cells was improved, but this was not judged to be statistically significant (Stackhouse et al., 2012). Tolerance to desiccation stress has been shown to provide cross-protection against UV irradiation, various sanitizing agents, dry heat, and bile salts (Gruzdev et al., 2011). This observation may, at least in part, explain why *Salmonella* can persist within the food production environment. Furthermore, some bio-molecules, including trehalose and sucrose can improve this bacterium’s ability to withstand desiccation (Gruzdev and Pinto, 2012). Dehydration at basic pH (8–10) can also enhance desiccation tolerance (Gruzdev and Pinto, 2012). Conversely desiccated bacteria were also found to be more susceptible to organic acids, a fact which may indicate a possible solution for the eradication of *Salmonella* from the factory environment (Gruzdev et al., 2011).

### INCREASED THERMAL RESISTANCE

The most intensely studied phenotype related to low-*a*_w_ stress is enhanced thermal resistance. Some of the earliest studies of this phenomenon were carried out over 30 years ago (McDonough and Hargrove, 1968; Dega et al., 1972) and research was focused on *Salmonella* in dried milk powders. McDonough and Hargrove (1968) investigated the dry heat tolerance of a cocktail of three *Salmonella* serotypes inoculated at 10^4^ CFU/g in non-fat dried milk. At 76.6°C which exceeds temperatures normally used for pasteurization, the bacteria were detectable after a 10-h period (McDonough and Hargrove, 1968). These findings highlighted the crucial role of moisture content in the effectiveness of heat destruction. At 4 and 7% moisture, there was insufficient killing even after 2 h at 85°C, however, increasing the moisture level to 25% a period of only 30 min was necessary to eliminate the bacteria (McDonough and Hargrove, 1968). Dega et al. (1972) reported that the percentage of milk solids also affected thermal resistance, and these authors observed that an increase in the solid content correlated with an increase in *z*-value. These authors also showed that *Salmonella* cultured at higher temperatures prior to heating were more resistant than those grown at lower temperatures (Dega et al., 1972). This observation may prove important to the PIF industry where ambient temperatures can reach high levels (up to 40°C) before the spray drying process (Mullane et al., 2007). Interestingly, *Salmonella* isolated from dry milk do not show any greater heat tolerance when exposed to pasteurization conditions in whole milk (Read et al., 1968). Similarly the heat tolerance of low-*a*_w_ food outbreak-associated *Salmonella* did not show any greater degree of heat tolerance when compared to other isolates. Stackhouse et al. (2012) observed that filamentous cells formed under NaCl stress actually have a reduced thermal tolerance to cells grown under non-stress conditions, which may lead to their reduced survival during processing, storage or preparation of a final product. Taken together, these facts suggest that these bacteria do not possess any features that specifically promote survival during heat treatment (Mattick et al., 2001).

In addition to the above, habituation at moderately low-*a*_w_ has been shown to significantly increase heat tolerance (Mattick et al., 2000a). The extent of this effect is also dependant on the humectant used. In media adjusted with glucose–fructose (*a*_w_ 0.95) there was greater than a fourfold increase in the *D*-value at 54°C (*D*_54_) after an incubation of 12 h, while a maximum increase of twofold in *D*_54_ was observed following incubation in media adjusted to *a*_w_ 0.95 with NaCl for 24 h or glycerol for 30 min (Mattick et al., 2000a). After this maximal increase had been achieved, continued incubation in the adjusted media correlated with a decrease in *D*_54_ value (Mattick et al., 2000a). Mattick et al. (2001) also demonstrated that the heat tolerance of cells in low-*a*_w_ media adjusted with glucose–fructose, NaCl or sucrose was increased at temperatures >70°C, whereas the opposite was observed for temperatures below 65°C. The nature of this information is crucial for food manufacturers, particularly in respect of the design of important lethality steps to be used during food production.

Numerous studies have reported on the heat tolerance of *Salmonella* in specific food products and these are extensively reviewed by Podolak et al. (2010). The most important conclusion that can be drawn from these investigations is that a general increase in heat tolerance is observed in low and intermediate-moisture foods, however, the extent of this varies between food products depending on other intrinsic and extrinsic properties. One other feature of inactivation in low-moisture foods is the observation of non-linear survival curves, often showing a concave-upward curvature. In these cases, it may be prudent to use the inactivation rate describing survival in the slower phase of the inactivation curve. Manufacturers should be aware of using published *D*- and *z*-values in order to develop heat inactivation models to derive and implement safety criteria in their production process. It is advised that they determine the heat tolerance of bacteria on a *case-by-case* basis (Podolak et al., 2010).

Finally, it is also important to remember that increased heat resistance at low moisture is not exclusive to *Salmonella* spp. The presence of water plays a crucial role in the killing effect of heat treatment, resulting from physicochemical interactions. An increase in thermal tolerance in reduced *a*_w_ has been documented in other bacteria such as *E. coli*, *Listeria monocytogenes*, *Saccharomyces* spp., *Lactobacillus plantarum*, *Torulopsis globosa*, *Bacillus* spp., and *Clostridium botulinum* (Murrell and Scott, 1966; Brown and Melling, 1971; Härnulv and Snygg, 1972; Gibson, 1973; Sumner et al., 1991; Laroche et al., 2005; He et al., 2011). But as mentioned previously, as *Salmonella* are the main pathogenic concern for food manufacturers of low-moisture products, the majority of thermal resistance studies carried out at reduced *a*_w_ have therefore focused on this species.

## SOURCES AND CONTROL OF CONTAMINATION

*Salmonella* is a ubiquitous organism in nature and as such can enter a production facility *via* a number of routes. Therefore, it is essential that manufacturers have effective control and monitoring procedures in place to track and trace *Salmonella*. Below, selected sources and implications of poor control of *Salmonella* are discussed.

### INGREDIENTS

The ingredients and raw materials used in any dry processing facility may be sourced from a variety of different suppliers and consist of relatively unprocessed items, such as raw milk or eggs. These materials may then be heavily processed in order to produce the end consumer product(s) according to agreed specifications. If an ingredient does not undergo any intervention treatment prior to entering the final food product, manufacturers must conduct suitable risk assessments of the relevant supplier to ensure that there is reduced risk of food-borne pathogens being present (Beuchat et al., 2011, 2013). As such, even a well-managed facility can fall victim to introducing *Salmonella* into the manufacturing environment and re-contaminating finished product due to poor sourcing, handling and choice of these raw materials.

In an outbreak involving eight cases of salmonellosis associated with baby cereal in 1995 in England (Rushdy et al., 1998), the implicated serotype was a *S.* Senftenberg which had previously been isolated from an undistributed batch of heat-treated bulk cereal received by the manufacturer in 1994. This batch was marked as unsatisfactory due to the presence of cleaning residues originating from milling machinery, however, no further investigative action into the supplier was conducted at the time. Following the reported cases, a re-evaluation of the Hazard Analysis and Critical Control Point (HACCP) system in place, later revealed that the same milling machinery was used for cereal that had not undergone a previous heat treatment, rendering this a potential source of cross-contamination. Control measures were implemented to prevent a repeat occurrence. This incident highlights the need for careful sourcing and monitoring of suppliers. The safety of a food product depends not only on the manufacturer but also on each one of the ingredients. There have been several outbreaks that resulted directly from a food manufacturer’s poor choice of raw ingredients. These include the manufacturing of a marshmallow product that contained raw egg and caused 36 cases of salmonellosis in 1995 (Lewis et al., 1996). In addition paprika-powdered potato chips and paprika powder were the cause of 1,000 cases of salmonellosis in Germany in 1993. In the latter case a variety of serotypes were involved and the resulting infections were associated with a relatively low infective dose (estimated to be less than 45 cells; Lehmacher et al., 1995). This particular outbreak emphasized the fact that even low numbers of cells adapted to dry conditions were capable of causing infection in humans (Lehmacher et al., 1995).

The reduction in pathogen load in wet raw ingredients is often the first step in microbial control. Pasteurization is one of the most common practices used to kill any vegetative bacteria present in liquid ingredients, such as milk. This is commonly accomplished by heating the liquid to 72°C for 15 s and is effective against enteric pathogens including *Salmonella* and *Campylobacter*. The conditions chosen for pasteurization are influenced by the solid content (dry content) of the product as well as its composition, consequently manufacturers need to assess protocols used for each raw ingredient requiring this pasteurization step (Beuchat et al., 2011). High-pressure processing (HPP) is an additional alternative technique for microbial control. Unlike heat treatments, HPP effectively inactivates microorganisms while causing only minimal changes to the organoleptic properties of the product (Torres and Velazquez, 2005). HPP has been shown as effective against *Salmonella* and acts uniformly on the product and with immediate effect (Torres and Velazquez, 2005). In addition to the above, irradiation is another method that can be applied for pathogen reduction in dry ingredients, such as spices, but its use is limited due to negative consumer opinion, although it is permitted by food regulators (Beuchat et al., 2011). Steam sterilization may be a suitable alternative.

Sometimes, the critical point of pathogen reduction in certain non-RTE foods, relies on the consumer applying appropriate cooking applications to assure the safety of the product. In 2007, a salmonellosis outbreak occurred from the consumption of frozen pot pies (CDC, 2007a). It was believed that the majority of the consumers incorrectly followed the microwave cooking instructions, which may have led to the consumption of under-cooked product (CDC, 2007a). The inclusion of validated cooking instructions that are clear and easy to follow on the packaging of such products is essential to reduce the risk of the consumer under-cooking products of this category.

### PERSONNEL

Personnel can be a major source of cross-contamination in the production environment (Greig et al., 2007; Todd et al., 2009). Improper hand washing, clothing and the presence of aerosols (from sneezing) and fomites are potential sources of pathogenic bacteria (Todd et al., 2009). Therefore all personnel should be fully trained in good manufacturing process (GMP) and must be aware of the negative implications concerning the general public when these guidelines are not adhered to. Beuchat et al. (2011, 2013) offer a number of recommendations to help prevent the entry of pathogens *via* this route. Health screening of personnel for pathogenic microorganisms in combination with a notification system to report food-borne illness should be taken into consideration (Beuchat et al., 2011, 2013). In order to reduce the risk of contamination in the final product, suitable clothing and footwear should be worn within the production area, without transfer to any other part of the facility (Beuchat et al., 2011, 2013).

Morita et al. (2006) conducted a study that investigated *Salmonella* cross-contamination in a Japanese oil meal factory. The manufacturing area was found to be highly contaminated in comparison to areas for receiving and storage of goods. In the manufacturing area, all operator footwear became contaminated with *Salmonella* 1 day after being disinfected. A similar 90% positive contamination rate was detected for the workers gloves. This paper stated that restricting movement of personnel between zones along with the regular disinfection of shoes is an important factor in limiting spread of bacteria to other parts of the production facility (Morita et al., 2006).

When structural plans are being designed for a dry product manufacturing facility, attention must be focused on suitable zoning practices. Wet and dry zones need to be recognized as well as the level of hygiene within those zones; basic or medium hygiene for wet areas and basic, medium, or high hygiene for dry zones, with separation within wet zones not being required to be as stringent as those in dry areas (Beuchat et al., 2011). A designated wet area would include the raw material processing zone, depending on the facility. Dry areas include packaging, storage, spray drying, and dry mixing, this area should be physically separated from all wet areas to reduce the introduction of moisture (Beuchat et al., 2011, 2013). Moreover, the movement of personnel should be controlled in these areas, with airlock rooms located between different hygiene zones for changing clothes/shoes. In compliance with this design, taps for hand washing should not exist within dry zones, due to the contamination risk they pose and the increase in humidity that they produce; disinfecting gels would be a suitable alternative (Beuchat et al., 2011).

### EQUIPMENT, WATER, AND AIR

The use of equipment in the manufacture of low-moisture foods that is not well designed and maintained, poses a significant cross-contamination risk. Crevices in machinery, flooring and walls, and dead ends in piping are potential areas for pathogen accumulation and subsequent contamination of the product. In 1985, a *S.* Ealing outbreak associated with PIF occurred in the UK with approximately 70 individuals being affected, the majority of whom were infants (Rowe et al., 1987). Contamination of the product occurred after cracks developed in the walls of the spray dryer. The bacteria entered the insulation material in the inner lining of the spray dryer and over time the accumulation of moisture and powder, along with high temperatures, provided *Salmonella* with a perfect habitat in which to grow and later contaminate the end-product (Rowe et al., 1987). In a similar manner an outbreak of *S.* Agona from a toasted oat cereal, occurred due to an inadequate design of the manufacturing environment that was subsequently discovered upon investigation. In this example, the processing machinery was open to the atmosphere (Breuer, 1999).

In a major outbreak of salmonellosis linked to peanut butter and peanut paste in the US in 2008–2009, more than 700 cases of illness were reported and the outbreak may have contributed to the death of nine individuals. In the FDA inspection report for one of the manufacturing plants implicated, a number of examples of bad practice were observed. These included failure to maintain equipment, containers, and utensils in a manner that protect against contamination, failure to clean productions lines after isolating *Salmonella* from finished product, failure to validate a critical control point, storing and handling raw materials and finished goods in the same area, failure to conduct cleaning and sanitizing operations to protect against food contamination and absence of a ventilation system for preventing cross-contamination (FDA, 2009).

In an outbreak of *S.* Infantis associated with dry pet food, it was observed that some equipment had cuts and gouges that presented difficulties for cleaning and sanitizing and could have possibly lead to harborage areas for *Salmonella* (CDC, 2012a). On the other hand, the facility in question also did not provide adequate hand washing facilities nor appropriate microbial testing of ingredients, therefore the route of contamination was difficult to ascertain (CDC, 2012a). However, this case highlights several aspects of hygiene practice failure which ultimately led the second documented human salmonellosis outbreak traced back to dry pet food (in the US; CDC, 2012a).

Improper cleaning and disinfection or the presence of leaking pipework may introduce moisture into the environment, an event which is usually avoided in low-moisture product production, as it significantly increases the risk of pathogen persistence and contamination (Beuchat et al., 2013). When moisture is permitted, bacterial cells that were previously metabolically inactive are given the opportunity to grow, when conditions are suitable and this development potentially leads to high levels of contamination in the production environment. The International Life Sciences Institute (ILSI) recommend that water should be limited in these areas or if required, well removed and segregated from the production area. The cleaning of any tools with water should be conducted in a designated area, far removed from the vicinity of the dry-area and non-potable water should never come into contact with the manufacturing site even if it is contained within pipework (Beuchat et al., 2011). If the use of water is unavoidable, it is suggested that both internal and external components of equipment should be cleaned but also the surrounding environment such as walls, ceiling, and floors and followed by drying and sanitization (Beuchat et al., 2011). This routine is carried out to prevent proliferation of bacteria that may have accumulated in these areas due to the introduction of moisture used to clean the equipment itself. Vacuum cleaners are commonly used in the dry cleaning of factories and these can also be used as an environmental sampling tool. Sand blasters using calcium carbonate provide an alternative to wet cleaning (Beuchat et al., 2011).

In addition to the scenarios discussed above, air is yet another vector by-which pathogens can contaminate the final product. In 2011, ILSI outlined a number of measures that should be considered with regards to air entering production floors (Beuchat et al., 2011). It may be necessary to include a positive pressure air system to prevent contaminated air (originating from a raw material storage zone) entering controlled production areas (Podolak et al., 2010). Filtering air that enters production zones may also prove effective. As an added useful measure to ensure that filters are effective, these should be cleaned and replaced on a regular basis and the system validated for removal of microorganisms (Mullane et al., 2008). This control measure would be particularly important if the air comes directly into contact with the food product. Morita et al. (2006) noted that dust particles in the air can contain *Salmonella*, thereby increasing the risk of cross- contamination.

### PEST CONTROL

As a final example, the control of pests in a manufacturing site is an integral part in the prevention of *Salmonella* cross-contamination. There are many pests that could act as a transmission vector for this bacteria, these are generally mobile and therefore measures to impede or restrict their movement throughout the production site should be considered and implemented where appropriate. A study by Pennycott et al. (2006) demonstrated that wild birds can carry a variety of different *Salmonella* serotypes, while Olsen and Hammack (2000) highlighted the common house fly (*Musca domestica*) a potential hazard.

In the investigation into the mechanisms of *Salmonella* cross-contamination in a Japanese oil meal manufacturing facility mentioned above a total of 41 rodents were captured. The mesentery lymph nodes and intestinal contents of all rodents were analyzed, and 46% tested positive for *Salmonella*, mainly those captured from highly contaminated areas (Morita et al., 2006). Further investigation determined that these isolates were identical to those recovered from the processing floor (Morita et al., 2006). Movement of rodents and other pests throughout the production facility, would be considered a serious contamination risk.

Although there seem to be no known cases of pests being identified as the direct source of *Salmonella* contamination in an outbreak related situation, the use of baits, traps, and screens to limit entry and movement is recommended (Podolak et al., 2010; Beuchat et al., 2011).

### MONITORING CONTROL MEASURES

Once control measures have been implemented in a production site it is necessary to monitor them on a regular basis to ensure that they are deployed correctly, to ensure the end-product is of a high safety standard and that it complies with defined criteria (Beuchat et al., 2011).

End-product testing is not the most suitable approach to confirm that manufactured goods are free of pathogen contamination. There are a number of reasons underlying this fact. Contamination may occur at such a low level that the organism may not be detected using analytical methods that are currently in place, therefore only highly contaminated lots would be identified. However, as stated above in some cases, low levels of bacteria are sufficient to cause widespread illness. Thus techniques with higher sensitivities may be required for such testing. Furthermore it is likely that the bacteria are distributed heterogeneously throughout the food matrix, again rendering end-product testing an unreliable method for monitoring control programs as only highly contaminated lots would be recognized (Beuchat et al., 2011, 2013). Expanding the number and size of samples tested along with thorough mixing may overcome this issue but increased testing costs and the practicality of large scale testing would also be a finance-limiting factor.

Perhaps the most efficient way to ensure control measures are being employed correctly is by the use of appropriate environmental monitoring programs (EMPs) of the production facility. EMPs are not control programs in themselves but rather a means of verification that other food safety measures in place are effective. A well-designed EMP will allow manufacturers to identify potential *Salmonella* sources and validate the efficacy of sanitation and zoning practices. The data that are then collected and reviewed on a regular basis will allow producers to rectify any problem that occurs before it becomes a product risk. In order to carry this out in an effective manner, an appropriate sampling plan must first be designed by those who fully understand the nature of the food product, the production process, and zoning controls that are in place throughout the plant (Beuchat et al., 2011). The Grocery Manufacturers Association (GMA) provide an online equipment design check list for low-moisture foods (GMA, 2010). If a positive sample is identified, corrective actions must be rapidly put in place to minimize any possible risks. These may include changes to cleaning practices, altering HACCP and GMP protocols, and modifications to equipment or processing area (floor, drains, etc.) design. Molecular sub-typing can be a powerful tool in tracking the source of pathogen contamination, for example, Morita et al. (2006) tested a variety of different locations throughout a production site and identified persistent strains through pulsed-field gel electrophoresis. This particular technique has also been effective in identifying critical control points related to *Cronobacter* in a PIF factory (Mullane et al., 2007). Monitoring the trends displayed by bacteria cultured from the manufacturing environment will help in the recognition of any patterns that may be emerging and ascertaining whether or not there may be a problem developing.

The use of effective recall teams in suspected product contamination incidents is also essential. For example, every year numerous peanut butter products are recalled due to suspected *Salmonella* contamination. It is important in these cases that an experienced recall team gather the information required to make the decision to recall a product, and communicate this to the relevant parties in an efficient and timely manner (IAFP, 2013). Following from this, appropriate measures to prevent subsequent contamination should be put in place. The majority of contamination results from poor sanitation, therefore it is important to ensure appropriate sanitation regimes, equipment design, and validation techniques are used to limit the persistence and spread of *Salmonella* in these factories (IAFP, 2013).

Frequent inspection, calibration and servicing of equipment as well as the organization of internal and external audits of the factory, for all suppliers of raw materials, will support the control measures that are being complied (Beuchat et al., 2011, 2013). The GMA in the US published a comprehensive guidance document for the control of *Salmonella* in low-moisture foods in 2009 (GMA, 2009). Examining the number of consumer complaints in conjunction with food-borne illness outbreak alerts could also alert manufacturers to a potential breakdown in their control programs (Beuchat et al., 2011, 2013).

## CONCLUSION

Low-moisture foods form an integral part of the modern human diet. *Salmonella* species are the most frequent pathogenic contaminants of such products and this is reflected in the number of cases of gastroenteritis occurring as a result of the consumption of low-moisture foods. Despite the fact that *Salmonella* cannot grow in low-moisture setting, these bacteria remain viable for extended periods of time and can cause infection when present at low levels in low-moisture foods.

The mechanisms used by *Salmonella* to survive long-term in low-moisture products and dry production environments are only now beginning to be described (Mattick et al., 2000b; Balaji et al., 2005; Deng et al., 2012; Gruzdev et al., 2012; Li et al., 2012; Finn et al., 2013a). These survival strategies may include but may not be exclusive to, filamentation of cells, the accumulation of osmoprotectant metabolites/molecules and switching to a metabolically dormant state. It is important that these survival strategies continue to be investigated to obtain a better understanding of mechanisms of survival under various low-moisture conditions applicable to industrial processes.

Several phenotypes have been associated with *Salmonella* in low-*a*_w_ environments. For example, salmonellosis resulting from consumption of a contaminated low-*a*_w_ product has been associated with a lower infectious dose. Exposure of *Salmonella* to low-*a*_w_ has been shown to confer cross-tolerance to other stresses including low pH, bile salt tolerance, resistance to disinfectants, UV irradiation, and heat.

A recent study attempted to ascertain phenotypic markers that could identify strains of *Salmonella* with the potential to pose a low-*a*_w_ hazard based on phenotypic profile (Finn et al., 2013b). While the study concluded that isolates originating from low-moisture environments showed decreased biocide susceptibility and higher tolerance to humectants in comparison to isolates from other origins, no distinct phenotypic markers were identified to determine source of origin (low-moisture versus other) which could cause a challenge for public health diagnostics.

In conclusion, in order for food manufacturers to develop the conditions to provide for a safe, *Salmonella* free food product they must implement a scientifically valid series of control measures. This includes monitoring raw ingredients and their suppliers, appropriate training of personnel, adequate zoning within the facility, and appropriate EMP. Lastly, correct design and maintenance of equipment, water and air supply systems, including appropriate cleaning and sanitizing regimes, are essential in limiting the risk of recontamination and cross-contamination.

## Conflict of Interest Statement

The authors declare that the research was conducted in the absence of any commercial or financial relationships that could be construed as a potential conflict of interest.
